# Patterns of Weight Change in Aging and Dying

**DOI:** 10.1093/geroni/igaa057.1587

**Published:** 2020-12-16

**Authors:** Stephen Thielke

**Affiliations:** University of Washington, Seattle, Washington, United States

## Abstract

Little research has characterized the natural history of weight change in older adults. Different changes may occur during aging and dying. We analyzed 18 years of weight measures from a cohort of 736,361 Veterans, all of whom had died at age 70 or older. We produced summary measures that accounted for both chronological age and number of years before death. Several clear population-level trends appeared. (1) The average weight of the sample declined across all ages at a rate of about 0.18 BMI points per year. (2) Starting about seven years before death, the amount of loss began to accelerate, reaching a decline of 0.75 BMI points in the year before death. (3) Changes in weight relative to years of remaining life were independent of chronologic age. People who died at age 70 experienced, on average, the same type and duration of terminal decline as did those who died at age 95. (4) The dying process involved a cumulative loss of about 1.3 BMI points. (5) The distribution of weights during advancing age both declined and narrowed. (6) Disproportionate deaths occurred at the lower BMI ranges (below a BMI of 24), and especially below 18, regardless of age. (7) The finding in #5 is explained by the entire cohort losing weight, with death of the thinnest members. These findings argue for examining survival time in studies of weight change. They indicate that weight loss may be a natural part of dying, rather than a risk factor for it.

